# Atrial Fibrillation Ablation with a Novel Fully 3D-Mapping-Integrated Multi-Electrode Radiofrequency Balloon Catheter

**DOI:** 10.3390/jcm13010207

**Published:** 2023-12-29

**Authors:** Yannick Teumer, Clemens Miesbichler, Andreas Hauke, Lyuboslav Katov, Carlo Bothner, Alexander Pott, Martin Müller, Benjamin Walter, Wolfgang Rottbauer, Tillman Dahme, Karolina Weinmann

**Affiliations:** 1 Department of Cardiology, Ulm University Heart Center, Albert-Einstein-Allee 23, 89081 Ulm, Germany; yannick.teumer@uniklinik-ulm.de (Y.T.); carlo.bothner@uniklinik-ulm.de (C.B.);; 2 Department of Cardiology and Angiology, Bonifatius Hospital, Wilhelmstraße 13, 49808 Lingen, Germany; 3 Department of Medicine I, Ulm University, Albert-Einstein-Allee 23, 89081 Ulm, Germany; martin.mueller@uniklinik-ulm.de (M.M.);; 4 Department of Cardiology, Angiology and Pneumology, Esslingen Hospital, Hirschlandstraße 97, 73730 Esslingen, Germany

**Keywords:** atrial fibrillation ablation, pulmonary vein isolation, radiofrequency balloon catheter, 3D mapping, single-shot catheter

## Abstract

Pulmonary vein isolation (PVI), as the cornerstone of atrial fibrillation (AF) ablation, has emerged a widely used therapy for patients suffering from AF. To improve PVI efficiency, single-shot catheters (SSCs) have been developed. Regrettably, SSCs are not integrated into 3D-mapping technology. In that regard, a novel radiofrequency balloon catheter (RFBC, Heliostar, Biosense Webster) with full integration into 3D-mapping technology has been developed. The aim of this study was to assess operative and follow-up outcomes of the RFBC in AF patients. In this monocentric prospective registry, patients with a first-time PVI using the RFBC were included. Follow-up visits were scheduled 3, 6, 12 and 24 months after ablation and in case of symptoms. A total of 171 patients (36.8% female) were included, with a mean age of 68.5 ± 10.2 years. Among them, 63 patients (36.8%) presented with persistent AF. Notably, no major periprocedural complications were observed. The mean follow-up period was 287 ± 157 days. In the Kaplan–Meier analysis, the estimated recurrence-free survival after 12 months was 81.8%. Based on our data, PVI with the fully 3D-mapping-integrated RFBC seems to be safe and effective and to have a favorable 12-month outcome in patients with paroxysmal and persistent AF.

## 1. Introduction

**Figure 1:**
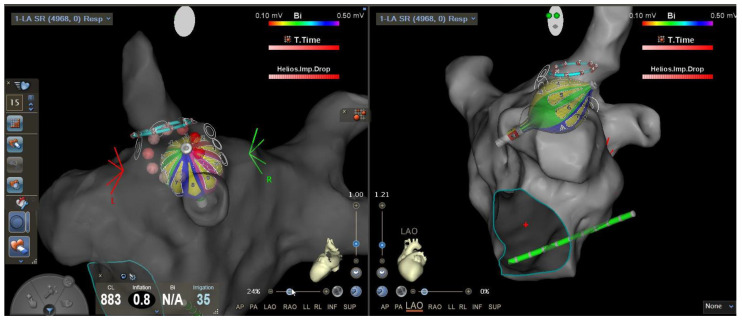
Visualization of the novel multi-electrode radiofrequency balloon catheter in the 3D-mapping system. The screenshot of the CARTO 3D-mapping system shows the multi-electrode radiofrequency balloon catheter (RFBC) in two different views.

Atrial fibrillation (AF) ablation has become a widely used therapy to treat AF [1]. Patients with symptomatic AF, particularly those suffering from heart failure, should be considered for AF ablation due to the benefits arising from maintaining sinus rhythm in this special population [2,3,4]. Consequently, the demand for pulmonary vein isolation (PVI), the cornerstone of AF ablation, is constantly growing. Continuous efforts are being made to advance ablation techniques to minimize pulmonary vein (PV) reconnection, reduce procedural duration and complications, and improve the reproducibility of the procedure. For this purpose, single-shot catheters have been developed. The most prominent variant of single-shot catheters is the cryo-balloon (CB), which has shown non-inferiority and shorter procedural duration compared to the point-by-point radiofrequency PVI [5]. The time-to-isolation (TTI)-guided titration of CB freeze duration has further reduced the procedural duration of PVI [6]. Unfortunately, to date, the CB or other true single-shot devices are still not integrated into 3D-mapping technology, resulting in the unavailability of substrate information with these catheters. Additionally, 3D-mapping integration can potentially reduce the fluoroscopy time needed for single-shot PVI. In that regard, a compliant, multi-electrode radiofrequency balloon catheter (RFBC, Heliostar, Biosense Webster, Irvine, CA, USA) has been developed (Figure 1) that is fully integrated into 3D-mapping technology (Carto, Biosense Webster, Irvine, CA, USA). Through this catheter, it is now possible to gather substrate information and to record pre- and post-ablation voltage information with a single-shot PVI catheter if desired. Furthermore, based on the possibility of adjusting the RF energy release of each electrode on the balloons’ surface, it is possible to titrate ablation energy depending on the PV proportion. For example, posterior-orientated electrodes were switched off earlier than anterior-orientated electrodes.

The aim of this prospective monocentric registry was to evaluate procedural and outcome data of the RFBC in paroxysmal and persistent AF.

## 2. Materials and Methods

### 2.1. Study Population

In this monocentric prospective registry, 171 consecutive patients with symptomatic paroxysmal or persistent AF were included between September 2021 and April 2023. Exclusion criteria were long-standing persistent AF, a left atrial diameter larger than 55 mm, and patients younger than 18 years. All patients underwent a first-time PVI-only procedure with the RFBC (Heliostar, Biosense Webster, Irvine, CA, USA) at our center (Ulm University Heart Center, Ulm, Germany). The data were prospectively collected as part of the ATRIUM registry (German Clinical Trials Register-ID: DRKS00013013). All patients provided written informed consent. The study complies with the Declaration of Helsinki and was approved by the local ethics committee at Ulm University.

### 2.2. Ablation Procedure

The initial patients included in this study had their procedures performed under deep sedation using midazolam, fentanyl, and propofol. Due to frequent pain-related excitation, the sedation protocol was changed to midazolam, remifentanil, and propofol [7]. All procedures were performed under uninterrupted oral anticoagulation. Antiarrhythmic drugs (AADs) were discontinued before to the procedure. Two right-sided femoral venous punctures were performed under sonographic guidance. A steerable diagnostic decapolar catheter (Inquiry, 6F, Abbott, Chicago, IL, USA) was placed in the coronary sinus for electrogram recording and temporarily placed into the superior vena cava for phrenic nerve pacing during energy delivery at the right-sided PVs. Transseptal puncture was performed under fluoroscopic and transesophageal echo guidance using a fixed transseptal sheath (CardiaGuide, 8,5F, Biosense Webster, Irvine, CA, USA) and a transseptal needle (HeartSpan, Merit Medical, South Jordan, UT, USA). An unfractionated heparin bolus was administered afterwards, targeting an activated clotting time between 300 and 350 s during the procedure. The fixed sheath was replaced by a steerable sheath (Guidestar, 13,5F, Biosense Webster, Irvine, CA, USA). Selective pulmonary vein angiography was accomplished with a 7F multipurpose or 6F pigtail catheter to identify all PV ostia via the steerable sheath. Subsequently, a circular mapping catheter (Lasso NAV, Biosense Webster, Irvine, CA, USA) was used to create an electroanatomic model (EAM) of the left atrium using a 3D-mapping system (Carto V7.2, Biosense Webster, Irvine, CA, USA). Afterwards, the RFBC with an intraluminal circular diagnostic catheter (LassoStar, Biosense Webster, Irvine, CA, USA) was introduced into the left atrium through the steerable sheath. After the LassoStar NAV became available in August 2022, mapping was performed with the RFBC and the intraluminal circular mapping catheter (LassoStar NAV, Biosense Webster, Irvine, CA, USA). The development of an ablation protocol was a dynamic process when the RFBC was only available to selected centers during the early evaluation phase. Balloon positioning and alignment to the PV was guided by the EAM and fluoroscopy. PV occlusion was guided by balloon electrode impedance and the injection of a contrast agent. For optimal balloon–tissue contact, a balloon inflation index > 0.8, and especially in the early study phase, an impedance of 100 ± 20 Ohm across all electrodes was targeted. Later, the focus for optimal balloon–tissue contact shifted from RF electrode impedance to the anatomical coaxial alignment of the balloon and the PV. Complete occlusion was followed by selecting two or three posterior-orientated balloon electrodes. RF energy (15 Watt) was delivered in a temperature-controlled manner to the posterior electrodes for 20 s and for 60 s to the remaining non-posterior balloon electrodes. During energy delivery, the PV electrograms were monitored on the intraluminal circular catheter for time-to-isolation (TTI) observation. TTI was defined as the time between the beginning of the energy delivery until the PVI was achieved. A PVI after a single energy delivery was defined as single-shot isolation (SSI). All PVs were checked for entry and exit blocks. The entry block was checked using voltage-mapping with the same catheters, which were used for initial mapping. A left atrial scar was defined as a voltage below 0.1 mV. The exit block was checked by pacing (20 mV, cycle length 500 ms) from the PV ostium with the intraluminal circular diagnostic or intraluminal circular mapping catheter. Prior to the energy delivery at the right superior pulmonary vein (RSPV), phrenic nerve pacing from the anterior-orientated balloon electrodes was performed at maximum output to evaluate proximity to the ablation site. In case of phrenic nerve capture, the balloon was repositioned, or single balloon electrodes were switched off during segmental energy delivery. In some cases, both maneuvers were performed. After catheter removal, a Z-suture was applied at the puncture site, and additional manual compression was performed for 5–10 min. A groin pressure bandage was applied for 6 h. Afterwards, the Z-suture was removed, and a groin pressure bandage was applied for additional 6 h. No protamine was used.

### 2.3. Esophageal Temperature Management

For esophageal temperature surveillance, a multi-electrode temperature probe (S-Cath, Circa Scientific LLC, Englewood, CO, USA) was placed transnasally into the esophagus at the level of the left atrium. No esophageal deviation was performed. If the esophageal temperature (ET) exceeded 39 degrees Celsius, the posterior electrodes were switched off earlier. If the posterior electrodes were already switched off and the ET was rising, the adjacent electrodes to the posterior electrodes were switched off too. If the ET exceeded 41 degrees Celsius, the energy delivery was completely stopped. If ET surpassed 42 degrees Celsius, esophagogastroscopy (EGD) was conducted within the next 7 days to detect thermal esophageal lesions (EDELs). EDELs were categorized according to the Kansas City Classification. Additionally, 40 mg pantoprazole per day was prescribed for 2 months. In the case of a detected EDEL, a re-EGD was performed 14 days later.

### 2.4. Periprocedural Management and Follow-up

All patients were monitored for 24 h after the procedure, including clinical examination, transthoracic echocardiography, and a 12-lead resting ECG. After PVI, outpatient clinic visits were scheduled for 3, 6, 12, and 24 months after the procedure, and in cases of symptomatic recurrence, including clinical examination, a 12-lead-rest-ECG and a 7-days-holter-ECG were performed. AT/AF recurrence was defined as every atrial tachyarrhythmia lasting longer than 30 s after a 3-month blanking period.

### 2.5. Study Endpoints

The efficacy endpoint was defined as AT/AF recurrence-free survival after 12 months according to the Kaplan–Meier analysis. Major complications were defined as any occurrence of periprocedural fatality, pericardial effusion with the need for pericardiocentesis, stroke, transient ischemic attack (TIA), myocardial infarction, persistent phrenic nerve palsy (PNP), atrio-esophageal fistula, and vascular access complications requiring specific treatment. Minor complications were defined as pericardial effusion without the need for pericardiocentesis, transient PNP, EDELs, and vascular access complications not requiring specific treatment.

### 2.6. Statistical Analysis

The statistical analysis was performed using SPSS Statistics (V29, IBM, Armonk, NY, USA). A *p*-value < 0.05 was considered significant. Categorial variables were described as frequencies and were analyzed with the Chi-square test or Fisher’s exact test as appropriate. Numeric variables were tested for normal distribution using the Shapiro–Wilk test and equal variance using Levene’s test. Normally distributed variables were described as mean ± standard deviation and were tested with Student’s *t*-test. Non-normally distributed variables were described as median ± interquartile range (IQR) and tested with the Mann–Whitney rank sum test. The recurrence-free survival was analyzed using Kaplan–Meier analysis. Differences in survival were tested with the log-rank test.

## 3. Results

### 3.1. Baseline Characteristics

A total of 171 consecutive patients (female 36.8%) were included in this study, with a mean age of 68.5 ± 10.2 years. Among them, 63 patients (36.8%) had persistent AF. The mean CHA_2_DS_2_-VASc score was 3.8 ± 2.0. Detailed patient characteristics are summarized in Table 1.

### 3.2. Procedural Characteristics

The median procedural duration was 88 min, with a median fluoroscopy time of 18.9 min. The median dwell-time of the RFBC in the left atrium was 23 min (Table 2). Single-shot isolation with the RFBC was possible in 70.6%. Detailed procedural data are summarized in Table 3. The mean TTI was 12.5 ± 6.7 s and was observed in 77.8% of all treated PVs. In 166 patients (97.1%), PVI was achieved with the RFBC only. In three patients, it was not possible to isolate the RSPV with the RFBC due to recurrent phrenic nerve (PN) capture from the balloon, despite recurrent balloon repositioning and a segmental ablation approach. In one patient, it was not possible to isolate the LSPV at the ridge despite multiple energy deliveries with the RFBC. In another patient, LIPV isolation was not possible with the RFBC due to recurrent esophageal temperature rise.

### 3.3. Safety

**Figure 2:**
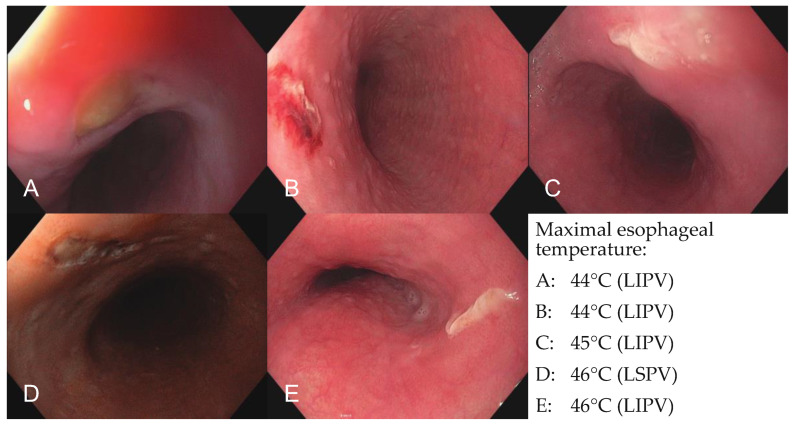
Esophagoscopic images of endoscopically detected esophageal lesions. Images of all five endoscopically detected esophageal lesions in patients with significant esophageal temperature rise (*n* = 35) during pulmonary vein isolation with the novel radiofrequency balloon catheter. (**A**) Type 2a lesion after esophageal temperature rise of 44 °C during ablation of the LIPV, (**B**) type 1 lesion after esophageal temperature rise of 44 °C during ablation of the LIPV, (**C**) type 2a lesion after esophageal temperature rise of 45 °C during ablation of the LIPV, (**D**) type 1 lesion after esophageal temperature rise of 46 °C during ablation of the LSPV, (**E**) type 2a lesion after esophageal temperature rise of 46 °C during ablation of the LIPV. LSPV = left superior pulmonary vein; LIPV = left inferior pulmonary vein.

No major complications and six minor complications (one transient PNP, five EDELs) occurred. The PNP resolved spontaneously within 48 h. An EGD was performed in 35 patients due to esophageal temperature rise as per the study protocol. In 5 of 35 patients, an EDEL was seen (Figure 2) in the esophagus. According to the Kansas City Classification, two lesions were categorized as a type 1 lesion (erythema without disruption of the mucosa) and three lesions were categorized as a type 2a lesion (superficial ulcers). In a re-EGD, all lesions resolved spontaneously without further sequelae. No atrio-esophageal fistula or vascular access complications occurred.

### 3.4. Outcome Data

**Figure 3:**
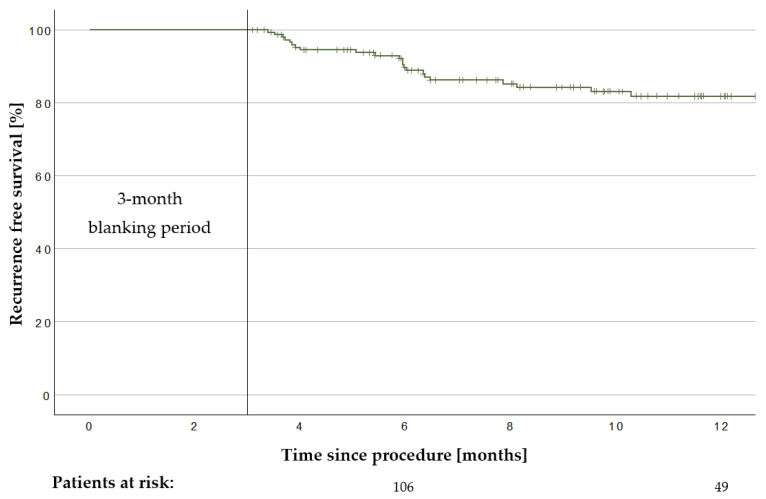
Recurrence-free survival after pulmonary vein isolation. Kaplan–Meier graph of the recurrence-free survival after pulmonary vein isolation with the novel multi-electrode radiofrequency balloon catheter after a 3-month blanking period in the overall group (*n* = 171). Recurrence-free survival after 12 months was 81.9% in the overall cohort.

**Figure 4:**
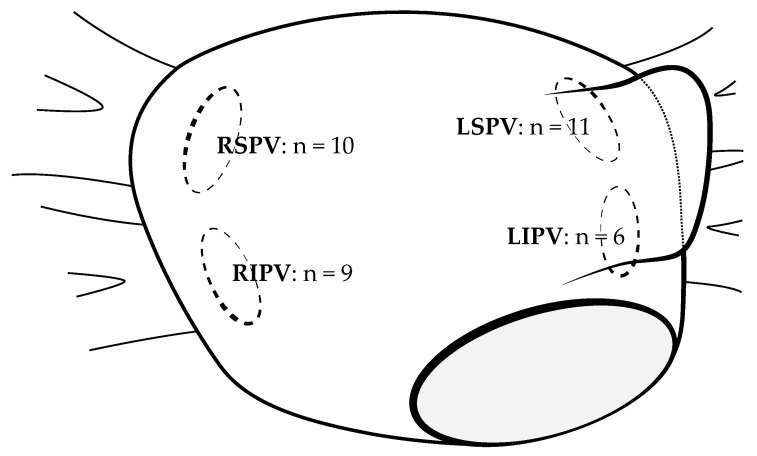
Distribution of the pulmonary vein reconnections. Overview of the reconnected pulmonary veins after first-time pulmonary vein isolation with the after first-time PVI with the novel multi-electrode radiofrequency balloon catheter detected in remapping procedures (*n* = 20). The number of reconnected pulmonary veins is depicted in a schematic illustration of a left atrium. LSPV = left superior pulmonary vein; LIPV = left inferior pulmonary vein; MV = mitral valve; RSPV = right superior pulmonary vein; RIPV = right inferior pulmonary vein.

The mean follow-up of the 171 treated patients was 287 ± 157 days. In total, 22 AT/AF recurrences were observed within the first 12 months. Twelve patients were on AADs after a 3-month blanking period. The Kaplan–Meier analysis estimated a recurrence-free survival of 81.8% after 12 months (Figure 3). Repeat 3D-mapping procedures were performed in 20 patients; 19 of them were driven by AF recurrence and 1 by atypical atrial flutter. In total, 36 PVs in these patients had been reconnected. The mean count of reconnected PVs was 1.8 ± 1.1 veins per patient. Figure 4 summarizes the locations of PV reconnection.

### 3.5. Subgroup Outcome Analysis

**Figure 5:**
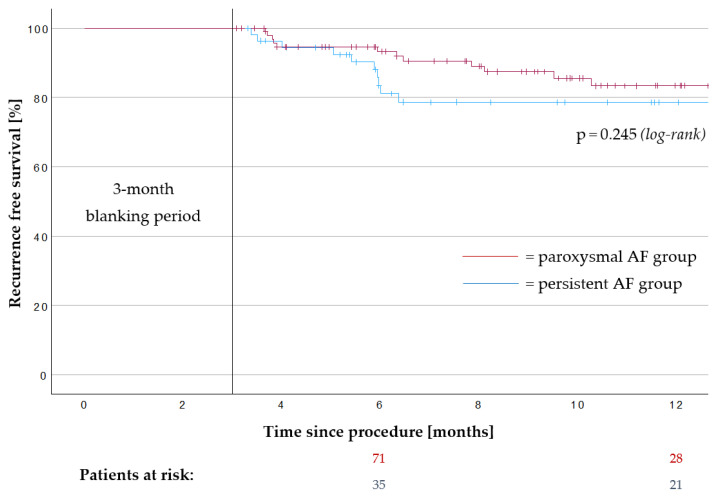
Recurrence-free survival after pulmonary vein isolation in the paroxysmal atrial fibrillation subgroup and the persistent atrial fibrillation subgroup. Kaplan–Meier graph of the recurrence-free survival after pulmonary vein isolation with the novel multi-electrode radiofrequency balloon catheter in the paroxysmal atrial fibrillation subgroup (*n* = 108) and the persistent atrial fibrillation subgroup (*n* = 63) after a 3-month blanking period. Recurrence-free survival after 12 months was 83.4% in the paroxysmal atrial fibrillation subgroup and 78.7% in the persistent atrial fibrillation subgroup. AF, atrial fibrillation.

In the paroxysmal AF subgroup, AT/AF recurrence occurred in 12 patients (11.1%) and it occurred in 10 patients (15.9%) in the persistent AF subgroup after 12 months. The Kaplan–Meier analysis estimated a recurrence-free survival of 83.4% in the paroxysmal AF subgroup and 78.7% in the persistent AF subgroup after 12 months (*p* = 0.245, Figure 5). Nine patients in the paroxysmal AF subgroup and three patients in the persistent AF subgroup were on AADs after a 3-month blanking period.

## 4. Discussion

To the best of our knowledge, this is the largest study to assess the safety, efficacy, and outcomes of AF patients treated with the RFBC. The RADIENCE study has shown PVI feasibility with the RFBC [8]. The SHINE study [9] and other publications [10,11,12] generated the initial evidence on acute efficacy and safety of the RFBC, but only limited data are available on RFBC-PVI outcomes [8,9,10,11]. Based on these data, the reported patient numbers are relatively small, and evidence concerning the treatment of persistent AF is sparse.

### 4.1. Safety

No major complications occurred in this study. There was no atrio-esophageal fistula, pericardial effusion, TIA, stroke or persistent PNP. One patient experienced a transient PNP. RF delivery was stopped immediately after loss of PN capture during RSPV isolation. The PNP resolved completely within 48 h. In this case, phrenic nerve pacing from the balloon was accidentally not performed prior to the energy delivery at the RSPV. The overall PNP rate of this study (0.6%) can be declared ‘low’ in comparison to other RFBC-PVI studies (0.7–3.3%) [8,9,10,11,12,13]. Interestingly, in these studies, the PNP rate seems to be lower if PN pacing from the balloons’ electrodes is performed before the energy delivery at the RSPV [8,9,10,11,12,13], just like in our study. This can be the reason for the low PNP rate in this study. Compared to the wide-area circumferential RF-PVI (0.17–0.48%) [14], the RFBC PNP rate seems to be similar. In comparison to the CB (4.1%), the PNP rate after RFBC-PVI seems to be lower [15]. One explanation, therefore, could be the previously mentioned possibility of evaluating the proximity of the PN to the ablation site by pacing from the balloons’ electrodes before the energy delivery at the RSPV. Thereby the opportunity exists to switch off single RF electrodes on the RFBC and relocate the balloon, which can lead to PNP risk reduction. Only a small amount of data are available concerning EDELs after RFBC-PVI [8,11,13]. In most studies, only a part of the cohort has undergone EGD. Moreover, the circumstances when an EGD was performed varies from study to study. That could be the reason why the EDEL rate after RFBC-PVI ranges between 0 and 8.0% [8,11,13] in the literature. In this study, an EGD was performed systematically, when the ET was exceeding 42 degrees Celsius, and an EDEL was detected in five patients (2.9%). In a re-EGD, all lesions resolved spontaneously without further sequelae. It is worth mentioning that in case of an ET rise, it was not uncommon that the ET was increasing rapidly. Even if the energy delivery was completely stopped at 41 degrees Celsius, the ET often climbed above 42 degrees Celsius. As a result, it is crucial to monitor the ET vigilantly and to react rapidly. Interestingly, in our cohort, EDELs were only seen in patients with an ET exceedance above 44 degrees Celsius. Surprisingly, no vascular access complications occurred. This can be caused by the sonographic guidance of femoral venous punctures and is not correlated to the RFBC, in our opinion.

### 4.2. Efficacy

The median procedural duration in this study was 88 min from skin to skin. Even if shorter procedural durations for RFBC-PVI under general anesthesia are described [9,11], this study is in line with the so-far published RFBC-PVI studies performed under deep sedation [8,9,10,13]. Compared to the CB-PVI, which is one of the most important representatives for single-shot PVI, the RFBC-PVI seems to be equally fast [6,16]. Surprisingly, there was no saving of fluoroscopy with the RFBC. Another cue for the single-shot character of the RFBC is the high SSI rate of 70.6%, which is competitive compared to the CB-PVI [16]. In contrast to the CB, acute PV isolation seems to be achievable more quickly with the RFBC documented in numerical shorter-mean TTIs (10–12.5 s versus 42–46 s) [6,9,10,11,12,13,16]. It is worth mentioning that the RFBC opens new opportunities in comparison to a standard single-shot catheter. Due to the full 3D-mapping integration and the possibility to switch off single RF electrodes on the balloon, 3D-guided RF lesions can be performed with a balloon catheter. Moreover, 3D guidance can be helpful in PVI patients with big common ostia. In these patients, there is need for a segmental PV ablation approach and no possibility for complete PV occlusion. In addition, 3D-guided posterior wall isolation with the RFBC is possible [17]. The recurrence-free survival of 81.8% after 12 months in this study is consistent with that reported in previous RFBC-PVI studies (72.2–86.4%) [8,9,11]. A similar 12 month recurrence-free survival is evident for the point-by-point RF-PVI and CB-PVI in the literature [6,16,18].

### 4.3. Subgroup Outcome

To the best of our knowledge, the persistent AF subgroup in this study is the largest treated with the RFBC so far [10,11,12]. In comparison to the paroxysmal AF subgroup, the baseline characteristics differ because of a bigger left atrial diameter (LAD) only. This can be explained by left atrial remodeling as the natural course of AF [19]. In addition, there was no significant difference between the procedural parameters of the paroxysmal or persistent AF subgroup. Although the RFBC was originally designed for the treatment of paroxysmal AF, our data suggest that patients with persistent AF can be treated successfully with the RFBC too. Interestingly, the estimated recurrence-free survival in the persistent AF subgroup after 12 months was only a little lower than in the paroxysmal AF subgroup. The relatively good outcome of the persistent AF subgroup might be explained by the fact that patients with a LAD bigger than 55 mm and long-standing persistent AF were excluded from this study.

## 5. Limitations

The current study reports data from consecutive patients in a high-volume center and is thereby limited by its single-center and non-randomized design. The approach of a 3D-mapping-integrated single-shot device is a new concept for achieving PVI. Workflow adaptations during the study may have an impact on the efficacy and procedural characteristics. The device in its first generation was restricted to highly experienced centers and operators only. The direct comparison to other non-3D-mapping-integrated single-shot catheters will show the additional benefit of this novel concept. Patients being screened for symptomatic AT/AF recurrence using 7-days-holter-ECG do not represent the actual AT/AF burden, and asymptomatic arrhythmia recurrence might be missed. Conclusions about the occurrence of atrio-esophageal fistulas are limited due to an overall low incidence of atrio-esophageal fistulas after PVI procedures and a relatively low number of patients in this context.

## 6. Conclusions

To the best of our knowledge, to date, this is the largest AF cohort treated with the novel RFBC for PVI. According to our data, PVI with the fully 3D-mapping-integrated RFBC seems to be safe and effective and to have a favorable outcome after 12 months in paroxysmal and persistent AF.

## Figures and Tables

**Table 1:** Baseline characteristics.

Baseline Characteristics	Total *n* = 171	Paroxysmal AF *n* = 108	Persistent AF *n* = 63	*p* Value
Age [years] (mean ± SD)	68.5 ± 10.2	67.8 ± 10.7	69.7 ± 9.3	0.245
Female, *n* (%)	63 (36.8)	41 (38.0)	22 (34.9)	0.691
BMI [kg/m^2^] (mean ± SD)	28.5 ± 5.5	28.2 ± 5.5	29.1 ± 5.4	0.134
CHA_2_DS_2_-VASc score (mean ± SD)	3.8 ± 2.0	3.7 ± 2.1	4.0 ± 1.9	0.439
Reduced ejection fraction ^a^, *n* (%)	69 (40.4)	41 (38.0)	28 (44.4)	0.405
Arterial hypertension, *n* (%)	129 (75.4)	77 (71.3)	52 (82.5)	0.099
Diabetes mellitus, *n* (%)	35 (20.5)	23 (21.3)	12 (19.0)	0.725
Prior Stroke or TIA, *n* (%)	23 (13.5)	15 (13.9)	8 (12.7)	0.826
CAD, *n* (%)	91 (53.2)	56 (51.9)	35 (55.6)	0.640
LAD [mm] (mean ± SD)	45.3 ± 6.3	44.3 ± 6.2	47.2 ± 6.0	0.003

AF, atrial fibrillation; BMI, body mass index; CAD, coronary artery disease; LAD, left atrial diameter; SD, standard deviation; TIA, transient ischemic attack, ^a^ left ventricular ejection fraction < 35%.

**Table 2:** Procedural characteristics.

Procedural Characteristics	Total *n* = 171	Paroxysmal AF *n* = 108	Persistent AF *n* = 63	*p* Value
Procedure duration (skin-to-skin) [min], median (IQR)	88 (45)	90 (43)	83 (45)	0.152
Dwell time [min], median (IQR)	23 (21)	23 (20)	22 (25)	0.400
Fluoroscopy time [min], median (IQR)	18.9 (15.6)	19.0 (15.7)	18.0 (12.7)	0.336

IQR, interquartile range.

**Table 3:** Pulmonary vein ablation data.

Ablation Data	Total *n* = 171	Paroxysmal AF *n* = 108	Persistent AF *n* = 63	*p* Value
Treated PVs (overall), *n*	669	420	249	-
LSPV, *n*	161	101	60	
Single-shot isolation (*n*, %)	128 (79.5)	79 (78.2)	49 (81.7)	0.600
TTI [s] (mean ± SD)	15.6 ± 8.6	14.5 ± 7.7	17.3 ± 9.6	0.149
RF applications (mean ± SD)	1.7 ± 1.3	1.7 ± 1.4	1.6 ± 1.0	0.676
LIPV, *n*	161	101	60	
Single-shot isolation (*n*, %)	125 (77.6)	76 (75.2)	49 (81.7)	0.345
TTI [s] (mean ± SD)	12.5 ± 6.2	12.4 ± 6.0	12.6 ± 6.6	0.788
RF applications (mean ± SD)	1.7 ± 1.0	1.7 ± 1.0	1.6 ± 0.9	0.131
RSPV, *n*	169	106	63	
Single-shot isolation (*n*, %)	114 (67.5)	72 (67.9)	42 (66.7)	0.866
TTI [s] (mean ± SD)	10.8 ± 5.4	10.4 ± 5.5	11.4 ± 5.4	0.088
RF applications (mean ± SD)	1.4 ± 1.1	1.4 ± 0.9	1.5 ± 1.4	0.846
RIPV, *n*	169	106	63	
Single-shot isolation (*n*, %)	99 (58.6)	64 (60.4)	35 (55.6)	0.538
TTI [s] (mean ± SD)	11.0 ± 5.0	10.6 ± 4.0	11.7 ± 6.2	0.880
RF applications (mean ± SD)	1.3 ± 0.6	1.3 ± 0.5	1.4 ± 0.7	0.213
RMPV, *n*	1	1	0	
Single-shot isolation (*n*, %)	1 (100)	1 (100)	-	-
TTI [s]	35 ^a^	35 ^a^	-	-
RF applications (mean ± SD)	1	1	-	-
LPV, *n*	8	5	3	
Single-shot isolation (*n*, %)	5 (62.5)	4 (80.0)	1 (33.3)	0.187
TTI [s] (mean ± SD)	11.7 ± 7.2	7.5 ± 0.7	20 ^a^	0.223
RF applications (mean ± SD)	3.0 ± 2.1	2.4 ± 2.2	4.0 ± 2.0	0.209

PV, pulmonary vein; LSPV, left superior pulmonary vein; LIPV, left inferior pulmonary vein; RF, radiofrequency; RSPV, right superior pulmonary vein; RIPV, right inferior pulmonary vein, LPV, left pulmonary veins with common ostium; SD, standard deviation; TTI, time to isolation ^a^ only one TTI was observed, as a consequence SD calculation is not reasonable.

## Data Availability

The data presented in this study are available on request from the corresponding author. The data are not publicly available due to data privacy law.
